# Effect of different thawing methods on the quality of mackerel (*Pneumatophorus japonicus*)

**DOI:** 10.1007/s10068-021-00966-0

**Published:** 2021-08-29

**Authors:** Peng-cheng Zhou, Jing Xie

**Affiliations:** 1 College of Food Science and Technology, Shanghai Ocean University, Shanghai, 201306 China; 2 Shanghai Professional Technology Service Platform on Cold Chain Equipment Performance and Energy Saving Evaluation, Shanghai, 201306 China; 3 National Experimental Teaching Demonstration Center for Food Science and Engineering, Shanghai Ocean University, Shanghai, 201306 China

**Keywords:** Mackerel, Thawing, Quality change, Microwave thawing, Flowing water thawing

## Abstract

Five thawing methods such as flow water thawing, ultrasonic flowing water thawing, air thawing, microwave thawing and low temperature thawing were used, and the physical, chemical properties and structure of mackerels after thawing were assessed. The results showed that the low temperature thawing had the best water retention, lower protein and fat oxidation. The microwave thawing had the shortest thawing time, but uneven heating leads to partial maturation. Air thawing prolonged exposure to air leads to high levels of protein and fat oxidation. The flow water thawing had better water retention than that of the ultrasonic flowing water thawing, only the thawing time was slightly longer than that of the ultrasonic flowing water thawing. In general, the low temperature thawing performed well after thawing. The flow water thawing used only 1/43 of the low temperature thawing's elapsed time after sacrificing some acceptable qualities. Thus, flow water thawing is more suitable for thawing frozen mackerel.

## Introduction

Mackerel (*Pneumatophorus japonicus*) is widely distributed in the Western Pacific Ocean (Tan et al., 2021). Because of its high nutritional value, low price and high yield, it has become an important economic fish in China (Wang et al., 2018). Mackerel contains trace elements such as calcium, phosphorus, iron, selenium and manganese, and tastes delicious, and the lipids are also rich in eicosapentaenoic acid (EPA) and docosahexaenoic acid (DHA), which can effectively prevent cardiovascular diseases.

Fish products deteriorate rapidly after death, however, freezing and frozen storage are one of the best ways to maintain the sensory and nutritional properties (Roiha et al., 2018). Freezing is the preferred method to solve the storage problem of aquatic products, so thawing before consumption is also very important for keeping its quality. Poor thawing methods will cause additional quality degradation of aquatic products. There were many different thawing methods, such as air thawing, water immersion thawing, ultrasonic thawing (Xiu-xia et al., 2021), microwave thawing, high voltage electric field thawing and high-pressure thawing (Jia et al., 2020). Each thawing method has its own advantages and disadvantages. After thawing, it leads to the loss of nutrients, fat and protein oxidation (Zhang and Ertbjerg, 2019).

At present, there are little researches on how to thaw frozen mackerels, so it is of great practical significance to explore the effect of thawing on mackerel. Different thawing methods have different degrees of damage to the quality of fish meat, which can be seen by indicators such as physicochemical properties, total volatile basic nitrogen (TVB-N) and Malondialdehyde (MDA). By comparing the pros and cons of various thawing methods, it can be judged that the best thawing method is suitable for domestic use.

The last part of the thawing cold chain, mainly operated by consumers, is limited by the thawing capacity of the family, and five suitable home thawing methods are chosen to explore the suitable thawing methods for consumers. The purpose of this study was to evaluate the effects of different thawing methods on the physical, chemical properties and structure of the mackerel after thawing. Thawing methods included flow water thawing (FT), ultrasonic flowing water thawing (UFT), air thawing (AT), microwave thawing (MT) and low temperature thawing (LTT).

## Materials and methods

### Sample preparation

Frozen mackerels were purchased from Shanghai Luchao Port. The mackerels weighed 215 ± 25 g and were transported to the laboratory within half an hour and stored in a refrigerator at − 18 °C.

### Materials

All chemical reagents and modified Bradford method protein concentration determination kit were purchased from Sangon Biotech (Shanghai, China). All these reagents were analytically pure. Malondialdehyde (MDA) assay kit (TBA method) was purchased from the Nanjing Jiancheng Bioengineering Institute (Nanjing, China).

### Thawing methods

The frozen mackerels were taken out of the refrigerator at − 18 °C and placed in a vacuum packaging bag. Five methods were used, namely flowing water thawing, ultrasonic flowing water thawing, air thawing, microwave thawing and low temperature thawing, respectively. The thermocouple probe (KeySight Agilent 34970A data collector, USA) was inserted into the center of the fish body to collect the temperature data of the fish body. In addition, considering that thermocouples cannot work under microwaves, the MT group does not use this temperature collector and has a separate digital thermometer (Fluke-NetDAQ32 Multipoint temperature collector). When the temperature of the fish body reaches 4 °C, the thawing ends.

#### Flowing water thawing (FT)

The sample was placed in a constant temperature water bath with a water pump to supply water, and the temperature of the water was controlled at 20 ± 1 °C.

#### Ultrasonic flowing water thawing (UFT)

The sample was put into the ultrasonic cleaner (SK5200HP Ultrasonic Cleaner, Shanghai Kedong Ultrasonic Instrument Co., Ltd., China), open the outlet of the machine, and use a water pump to provide a steady flow of water at both temperature and speed. The power of ultrasonic cleaner is 200 W and the temperature is controlled at 20 ± 1 °C.

#### Air thawing (AT)

The sample was put into a constant temperature and humidity box (BPS-100CL constant temperature and humidity box, Shanghai Yiheng Scientific Instrument Co., Ltd, China), the temperature is controlled at 20 ± 1 °C.

#### Microwave thawing (MT)

The sample was put in a microwave oven (NN-GD568 2 450 Hz Microwave Oven, Panasonic Shanghai Microwave Oven Co., Ltd., China) with a thawing power of 400 W.

#### Low temperature thawing (LTT)

The sample was thawed in a 4 °C constant temperature refrigerator (KK25F55TI refrigerator, Siemens AG, Germany).

### Determination of physicochemical properties

#### Thawing time

The thawing time was recorded every 10 s until the central temperature of the sample reached 4 °C.

#### Thawing loss

The mackerel was weighed with a balance before thawing and its weight the mass *m*_1_ is recorded. After thawing, the mackerel was dried with paper, re-weighed, and the mass *m*_2_ was recorded. The thawing loss was calculated according to the following equation:

1 $$ {\text{Thawing}}\,{\text{loss}} \left( \% \right) = \frac{{m_{1} - m_{2} }}{{m_{1} }} \times 100\% $$

#### Cooking loss

The back meat of skinless fish of about 2 g was taken and the mass of *m*_3_ was recorded. The sample was put into a polyethylene ziplock bag, boiled in a water bath at 85 °C for 20 min and then taken out, cooled to room temperature naturally, wiped off the surface moisture with kitchen paper, weighed again, and recorded the mass as *m*_4_. The cooking loss was calculated according to the following equation:

2 $$ {\text{Cooking}}\,{\text{loss}}\left( \% \right) = \frac{{m_{3} - m_{4} }}{{m_{3} }} \times 100\% $$

#### Water-holding capacity

The skinless fish back meat with a size of 1 cm × 1 cm is cut off, and its weight is recorded as *m*_5_. The sample is wrapped with double-layer filter paper and placed in a centrifuge tube, and centrifuged at 3000 × g for 10 min at 4 °C. At the end of centrifugation, the fillets were taken out and weighed and recorded as *m*_6_. The water-holding capacity was calculated according to the follows:

3 $$ {\text{Water}}\,{\text{holding}}\,{\text{capacity}}\left( \% \right) = \frac{{m_{5} - m_{6} }}{{m_{5} }} \times 100\% $$

#### Color measurement

The color of the fish was measured by a chroma meter (CR-400, Konica Minolta, Tokyo, Japan), and the three values displayed by the chroma meter, L* (lightness), a* (redness/greenness) and b* (blueness/yellowness). Five different areas were measured on the slice surface.

#### Texture

Thawed mackerel was cut into 2.0 cm × 2.0 cm × 1.0 cm cubes and measured by a texture analyzer (TMS-PRO Tissue Instrument, FTC Corporation, USA). P/6 flat bottom cylindrical probe was used. The pre-measurement rate was 3 mm/s, the test rate was 1 mm/s, the shape variable was 50%, and the return distance was 20 mm. The samples were measured 5 times in parallel for each group.

#### Electro conductivity

2 g of minced fish meat was put into a centrifuge tube, and 20 ml of deionized water was added to the tube. The solution was mixed and allowed to stand for 30 min, and the supernatant was filtered. The filtrate was measured with a conductivity meter (METTLER TOLEDO FE30, Zurich, Swiss), and the data was recorded.

#### Total volatile base nitrogen

A Kjeldahl nitrogen analyzer (Kjeltec 8400, Foss, Denmark) was used to determine TVB-N (Total volatile base nitrogen). After modification, 5 g of minced fish and 1 g of magnesium oxide were taken for detection.

#### Malondialdehyde

2 g minced fish meat and 18 mL physiological saline were added to the centrifuge tube. After being fully homogenized, the tube was placed in a 4 °C centrifuge at 6000 × g for 10 min to extract the supernatant. According kit (Malondialdehyde (mda) assay kit (TBA method), Nanjing Jiancheng Bioengineering Institutel, China), the absorbance value of 1 cm diameter at 532 nm was measured. MDA was calculated using the formula given by assay Kit.

#### Low-field nuclear magnetic resonance (LF-NMR) and magnetic resonance image (MRI)

The sample was cut into 3 cm × 3 cm × 0.5 cm (approximately 5 g) and sealed with polyethylene plastic wrap. The sample was placed in LF-NMR analyzer (MesoMR23-060H.I, Niumag Corporation, Shanghai, China) measurement channel, and the channel had a respective 21 MHz frequency stabilized by CPMG pulse. LF-NMR acquisition parameters are as follows: a receiver bandwidth frequency (SW) = 200 kHz, the analog gain (RG1) = 20 DP, P1 = 19.00 s, digital gain (DRG1) = 3, TD = 200,074, the gain of the preamplifier (PRG) = 1, the interval (TW) between consecutive scans = 3500 ms, number of scans (NS) = 8, P2 = 38.00 s echo time (TE) = 1.000 ms, NECH = 1000. Proton density weighted image parameters as follows: repetition time (TR) = 500 ms, TE = 18.2 ms.

#### Microscopic observation

The thawed fish was cut into 3 mm × 3 mm × 2 mm slices and fixed with formalin solution with a volume fraction of 5% for 24 h. Ethanol solution was used for gradient dehydration, then xylene was used for transparency and finally paraffin-embedded. Paraffin sections with a thickness of 10 μm were sectioned. After staining, the samples were observed and photographed by biological microscope (Eclipse E200 Biological Microscope, Nikon Instruments Ltd., Japan).

### Data analysis

Except where noted, the experiment all has three parallel. IBM SPSS Statistics 22 software was used for one-way ANOVA and Origin 2021 software was used for drawing.

## Results and discussion

### Thawing time

**Fig. 1:**
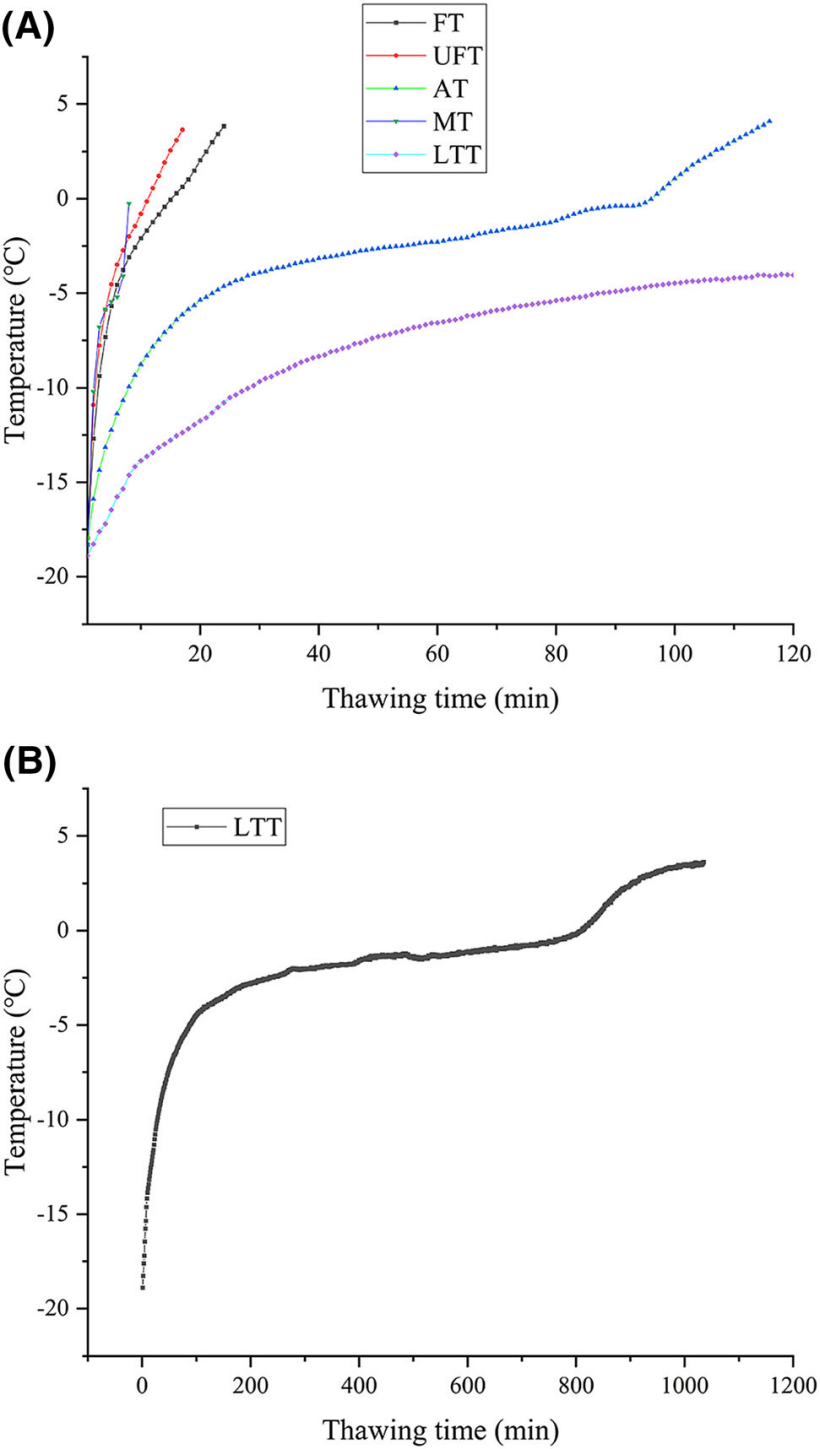
Thawing curves (**A**) (FT: flow water thawing, UFT: ultrasonic flowing water thawing, AT: air thawing, MT: microwave thawing and LTT: low temperature thawing), thawing curves (**B**) (LTT separate temperature curve)

The order of thawing time from fast to slow was low temperature thawing (4 °C), air thawing (20 ± 1 °C), flowing water thawing (20 ± 1 °C), ultrasonic flowing water thawing (20 ± 1 °C), microwave thawing (400 W), and the time required was 1035, 116, 24, 17 and 8 min, respectively. The thawing curves are shown in Fig. 1(A), where the LTT thawing time is much longer than the other thawing methods and is therefore shown separately in Fig. 1(B). Microwave thawing took the shortest time because the microwave can quickly heat frozen mackerel (Wang et al., 2015). However, due to the spindle structure of mackerel, the center of the fish was not thawed, and the tail was already matured. Low temperature thawing needed more time because the temperature difference between the thawing environment and the mackerel was small, resulting in slow heat transfer. The thawing time of the UFT group was shorter than that of the FT group. This situation was because the ultrasound itself carried energy, which could accelerate the melting of ice crystals and shorten the thawing time (Cai et al., 2019b).

### Thawing loss, cooking loss, water-holding capacity

The thawing loss, cooking loss and water-holding capacity caused by different thawing methods are listed in Table 1. After the samples were frozen and thawed, their cooking losses increased and their water holding capacity decreased. The thawing loss of MT was significantly higher than those of the other groups, the cooking loss was slightly higher than those of the other groups, and the water holding capacity was lower than those of the other groups. This may be because microwave thawing destroyed the properties of the protein, and ice crystals cannot bind to the protein after melting. Similar findings were found when Xia et al. (2012) studied different ways to thaw the longest muscle of pigs, with microwave thawing leading to more thawing losses and cooking losses, resulting in reduced taste and nutritional loss of the samples. In addition, after microwave thawing, the tail of the fish will mature and increase the loss of thawing (Baygar and Alparslan, 2015). Although it took a short time for MT to thaw, the water-holding capacity of mackerel was drastically reduced after thawing. LTT had the smallest thawing loss and cooking loss, and the water holding capacity is the largest. This may be because low temperature thawing process gentle and the ice melts slowly, reducing the denaturation of proteins and muscle tissue to absorb the melting ice crystals (Javadian et al., 2013).

**Table 1:** Through different thawing methods, the thawing loss, cooking loss and water retention capacity of mackerel

Thawing treatments	Thawing loss (%)	Cooking loss (%)	Water-holding capacity (%)
Fresh	–	18.48 ± 0.84^d^	74.52 ± 83^a^
Flow water thawing	2.64 ± 0.35^b^	20.77 ± 0.34^c^	68.24 ± 0.97^c^
Ultrasonic flowing water thawing	2.37 ± 0.06^b^	20.59 ± 0.44^c^	69.77 ± 2.03^ cd^
Air thawing	2.30 ± 0.15^b^	21.22 ± 0.28^b^	70.06 ± 1.34^ cd^
Microwave thawing	5.85 ± 0.09^a^	22.63 ± 0.77^a^	63.18 ± 2.78^d^
Low temperature thawing	1.28 ± 0.02^c^	19.86 ± 0.56^c^	73.75 ± 1.85^b^

Values represent means ± standard deviation. Different letters in the same column indicate significant difference (*P* < 0.05)

Compared with FT, UFT had better water retention, because ultrasound could reduce the damage to myofibril structure caused by thawing, while maintained the integrity of muscle fibers, thereby enhanced the water retention capacity of fish (Li et al., 2019b). The energy carried by ultrasonic waves could be absorbed by the sample during the thawing process, which accelerated the melting of ice crystals and reduced the loss of water (Wang et al., 2021a). However, this increase in UFT was not significant, probably because of the limitation of the effect of single-frequency ultrasound on thawing. Based on this, Wang et al. (2021b). found that dual-frequency sequential ultrasound had a better effect on thawing of small yellowtail. The AT group did not perform much in terms of water retention, which was similar to the FT and UFT group.

The cooking loss is due to the thermal denaturation of myofibrils, the destruction of muscle structure, and the exudation of a large amount of juice and soluble substances. The cooking loss has the same trend as the thawing loss, which is consistent with the findings of previous studies (Sun et al., 2019). Some nutrients in fish meat lost with the precipitation of water (Oz and Zikirov, 2015). Obviously, low temperature thawing can keep the moisture in the fish very well, and can well retain the original nutrients of the fish. Microwave thawing had a poor performance in water retention, and the loss of nutrients was the most serious.

### Color

The color of the fish is a direct reflection of the quality of the fish (Purslow et al., 2020), and the color changes of the fish depend on thawed methods. Frozen storage reduces the L* value of fish muscles, and a high level of L* value reflects a good thawing method. It could be seen from Table 2 that the L* value of LTT was significantly higher than those of other groups (*P* < 0.05), which was closest to the color of fresh samples. For the b* value, there were slight differences among the different thawing methods, but no significant differences in the a* value. The MT group had the worst color perception, which may be caused by fish ripening and protein denaturation caused by high temperature during microwave thawing. This was similar to what has been reported by Genc et al. (2015), the fish had better brightness after thawing in the refrigerator. However, there were also some differences between his study and ours, Genc found that there was a slight difference in a value but no difference in b value through different thawing methods. The reason for this difference may be the different species of fish. In general, the color of frozen mackerel becomes darker after thawing because of the loss of water and damage to the microstructure (Zhang et al., 2021).

**Table 2:** Color of mackerel muscle thawed by different thawing methods

Color	L*	a*	b*
Fresh	44.17 ± 0.45	6.49 ± 0.63	4.76 ± 0.38
Flow water thawing	39.13 ± 0.87^c^	5.07 ± 1.3^a^	4.02 ± 1.2^a^
Ultrasonic flowing water thawing	39.51 ± 0.95^c^	3.8 ± 0.92^a^	2.55 ± 0.39^ab^
Air thawing	40.95 ± 0.65^b^	3.73 ± 1.82^a^	3.12 ± 0.76^ab^
Microwave thawing	35.34 ± 0.36^d^	2.64 ± 0.34^a^	1.36 ± 0.25^b^
Low temperature thawing	42.76 ± 0.91^a^	4.47 ± 1.31^a^	3.12 ± 1.04^ab^

Values represent means ± standard deviation. Different letters in the same column indicate significant difference (*P* < 0.05)

### Texture

**Fig. 2:**
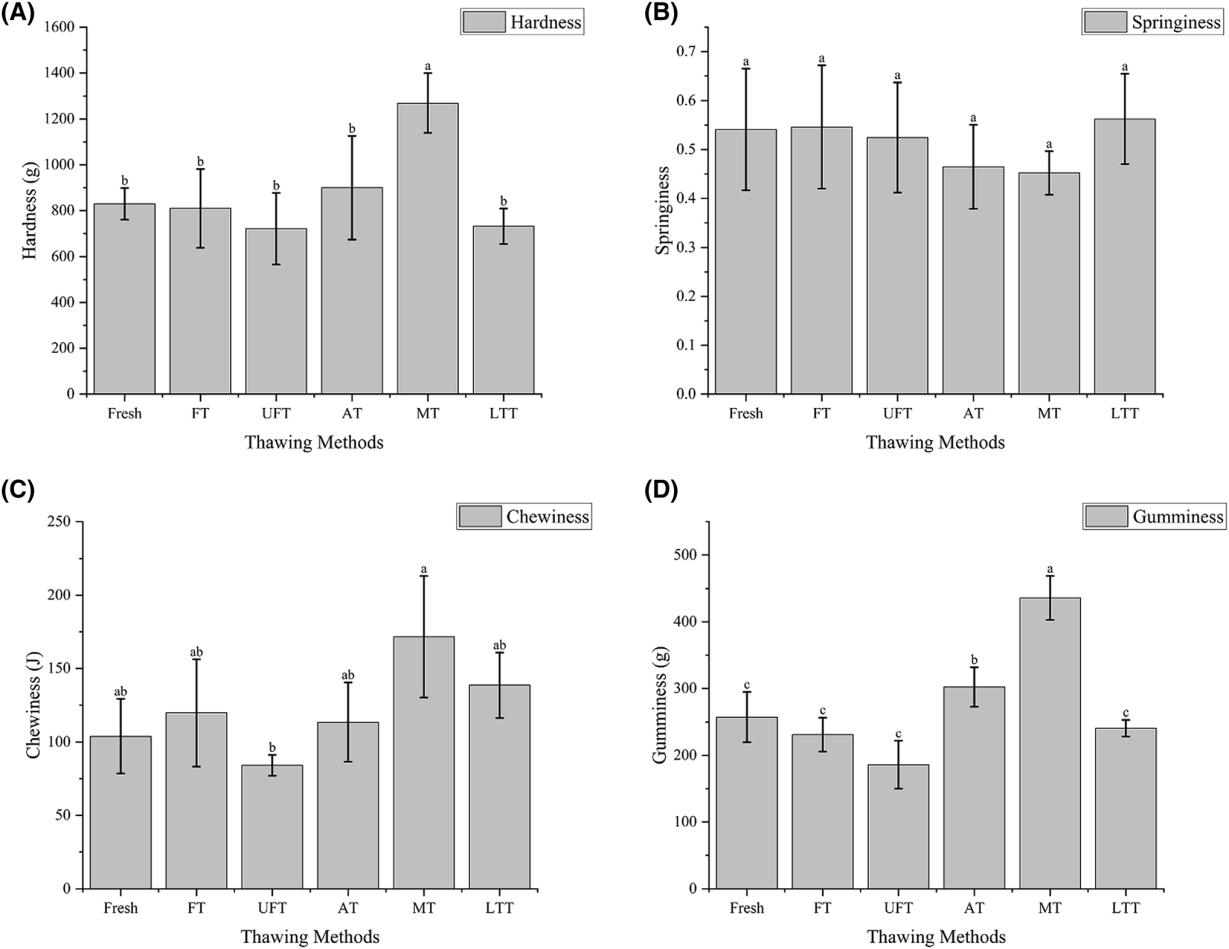
Effect of difference thawing methods on texture of mackerel of hardness (**A**), springiness (**B**), chewiness (**C**) and gumminess (**D**)

After being frozen and thawed, the texture of mackerel deteriorates (Xia et al., 2012). The results of texture analysis are shown in Fig. 2. In this figure, A, B, C and D indicate the mackerel's hardness, springiness, chewiness and gumminess, respectively. Fish fiber texture is mainly due to the state of the muscle tissue. Hardness indicates the firmness of fish, and the thawing method has a significant effect on the hardness. From Fig. 2(A), the hardness value of the MT group was significantly higher than that of fresh samples and other groups (*P* < 0.05), and there was no significant difference in the hardness value of the other groups, which may be caused by overheating in the microwave caused the oxidation and aggregation of fish protein (Wang et al., 2020a). Cai et al. (2020) also found a similar phenomenon after thawing frozen largemouth bass. The hardness of microwave thawed fillets is harder than other thawing methods. Some studies had found that the proper ultrasound power could increase protein solubility and increased the water content in the muscle, causing a decrease in hardness (Guo et al., 2021). Figure 2(B) showed that there was no significant difference in springiness of all groups, indicating that the thawing method had little influence on springiness C and D indicated that compared with other groups, the UFT group had lower chewiness and gumminess. It might be that the mechanical shock caused by cavitation changed the structure of the fish (Li et al., 2019a). After thawing, the textural characteristics of LTT were closer to those of fresh samples. FT and AT and LTT had similar textural characteristics, excluding Gumminess which was somewhat higher, with no significant differences, and the texture of all three groups of samples after thawing was between that of MT and UFT.

### Electro conductivity

**Fig. 3:**
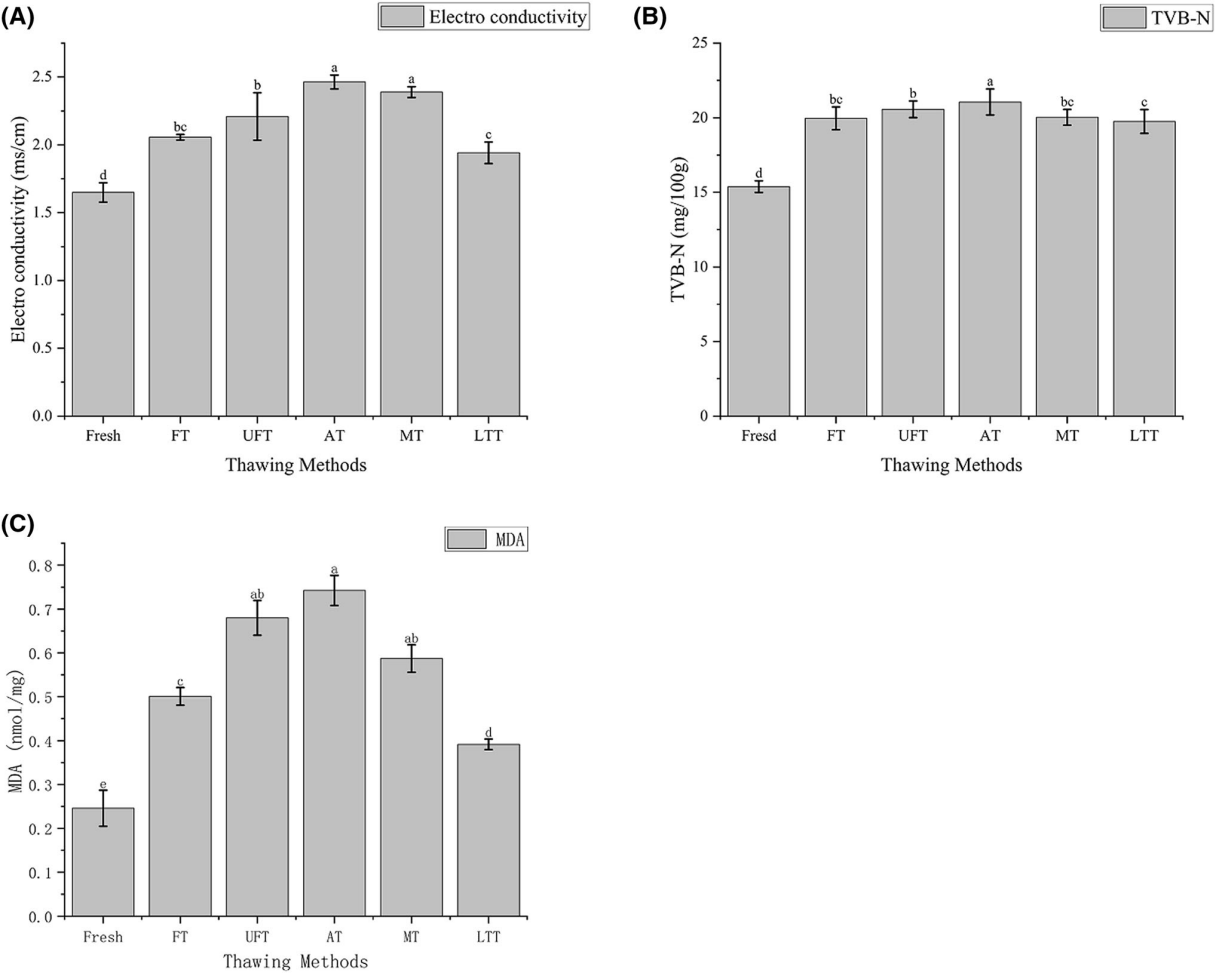
Effects of different thawing methods on electro conductivity (**A**), TVB-N (**B**) and MDA (**C**) of mackerel

The difference in thawing methods causes fish protein and fat to be decomposed into small molecular substances under the action of exogenous microbial protease, which existed in ionic form and enhanced the conductivity of the solution, resulting in enhanced conductivity of the fish extract (Shirsat et al., 2004). Therefore, the conductivity can reflect the freshness of the fish, and the conductivity is inversely proportional to the freshness. The value of electro conductivity is shown in Fig. 3(A). The conductivity of the fresh samples increased after freezing and thawing. The conductivity of AT was the largest, followed by MT, and LTT was the smallest. There was no significant difference between FT and UFT, so the effect of FT and UFT on conductivity was minimal. The reason for this may be that the AT group had more opportunities to contact the air, which caused more conductive substances to be decomposed, and the MT group brought a lot of heat in the thawing process, which increased the electrolyte ionization degree. The LTT group had a low degree of contact with oxygen in a low temperature environment, so it produced a small number of ions and low conductivity.

### Total volatile base nitrogen

TVB-N is a useful index for evaluating the freshness of aquatic products (Wang et al., 2020b). Through different thawing methods, the TVB-N value of mackerel changes are shown in Fig. 3(B). The TVB-N values of all thawed mackerel increased, and the lower the TVB-N value, the fresher the fish product. The LTT group had the lowest TVB-N value. It may be that the lower temperature of the LTT group has less effect on the protein. The TVB-N value of the other four groups was significantly lower than that of the AT group. It may be that the AT group had more time to contact with the air, which led to an increase in protein degradation and increased production of ammonia and amines (Mousakhani-Ganjeh et al., 2015). The longer thawing time in the AT group at the same time provided conditions for microbial activity and promoted the degradation of soluble substances (Lan et al., 2021). The difference in TVB-N value of FT, UFT and MT group was small, and these three thawing methods had no obvious influence on the TVB-N value of mackerel.

### Malondialdehyde

The body of mackerel produces lipid peroxides such as MDA during the lipid oxidation process (Wang et al., 2016). Testing the content of MDA can reflect the degree of lipid peroxidation in the body to understand the degree of oxidation in the fish body. Figure 3(C) shows the MDA content of mackerel with different thawing methods. The higher the degree of fat oxidation, the higher the amount of MDA. The MDA content of fresh samples spiked after freezing and thawing. The highest MDA content in the AT group may be due to the fact that fish meat has more opportunities to be exposed to oxygen, resulting in a high degree of fat oxidation. Javadian et al. (2013) made similar findings when thawing frozen rainbow trout, with samples thawed in air showing the highest level of fat oxidation. UFT and MT groups generated extra heat during the thawing process and accelerated the oxidation reaction between protein and fat. Some studies point out that the highest TBA values were found in microwave thawing because the high energy generated by microwaves stimulated the oxidation of fat (Ersoy et al., 2008). The FT group had a lower oxidation level because it was thawed in water and isolated from oxygen. The LTT group was in a closed environment in a low temperature environment with the lowest degree of oxidation, so the MDA content was significantly (*P* < 0.05) lower than those of other groups. In general, the LTT group achieved lower fat oxidation after thawing. It was worth mentioning that the FT group achieved a good thaw performance, which exceeded expectations.

### LF-NMR and MRI

**Fig. 4:**
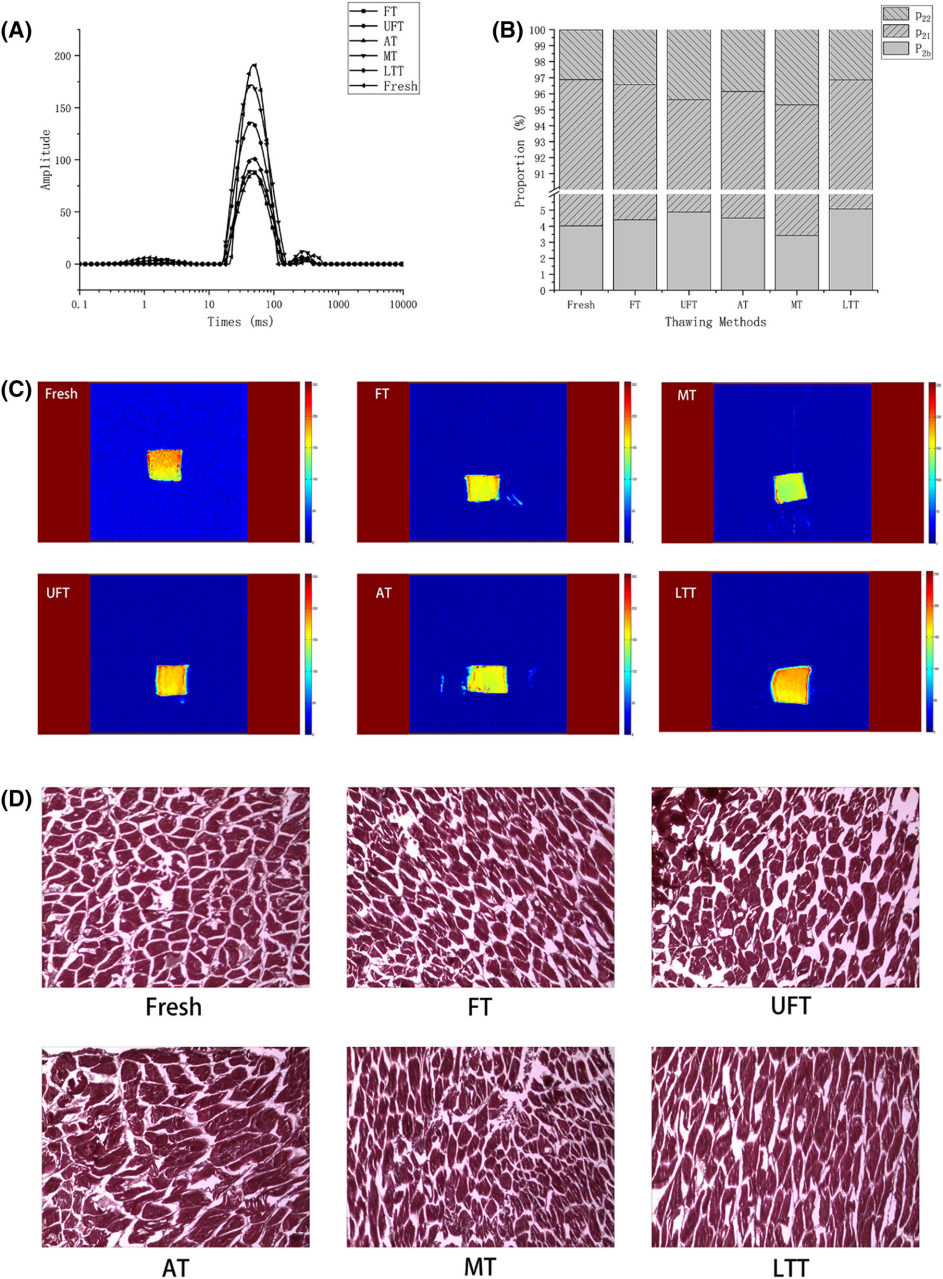
Effects of different thawing methods on the transverse relaxation time (**A**), distribution of water (**B**), magnetic resonance image (**C**) and microstructure (× 40) of mackerel

The water in the muscle can be distinguished into three forms, namely, bound water, immobilized water, and free water. Efficiently by a low-field NMR detection food matrix water migration, changes in proton relaxation behavior reflect the relationship between water and meat tissue structure (Wang et al., 2020c). Through different thawing methods, the transverse relaxation time T_2_ of mackerel is shown in Fig. 4(A). The peak value between 0 and 10 ms (T_21_) indicates bound water. The peak value between 10 and 100 ms (T_22_) is representative of the main peak immobilized water. The peak value between 100 and 1000 ms (T_23_) represents free water. Figure 3(B) shows the distribution of water in thawed mackerel muscles in different ways. As seen in Fig. 4(B), the MT group had more free water (T_23_) than the fresh samples and the other groups (*P* < 0.05), indicating that under this thawing method, the water in the fish tissue was easy to migrate out. This situation was because the structure of myofibrillar protein was severely damaged, resulting in the conversion of non-flowing water into free water (Qian et al., 2019). The LTT group had the lowest free water content and was closest to the fresh samples. The bound water content was significantly higher than those of the other groups (*P* < 0.05), indicating that this thawing method had the best water retention, which was consistent with the results of the study in “Thawing loss, cooking loss, water-holding capacity” section.

Figure 4(C) is a magnetic resonance image of each group. The red part of the pattern represented by high proton density, and blue indicates low proton density (Wang et al., 2020d). The higher the density of red in the pattern, the more water there is in the fish (Zhu et al., 2021). It can be directly seen from Fig. 3(C) that the MRI of fresh samples had the highest red brightness, and the MRI of thawed mackerel fish had the highest to lowest red brightness in the order of LTT, UFT, FT, AT, and MT, which was consistent with the water retention study.

### Microscopic observation

After thawing, the ice crystals can damage the structure of muscle tissue (Cai et al., 2019a). As can be seen in Fig. 4(D), the difference between the LTT group and the fresh samples was the smallest, which indicates that LTT reduced the effect of ice crystals on the muscle, and the muscle tissue was more complete and the muscle fibers were arranged in a more orderly manner. The AT group was slightly sparser than that of the LTT group, which may be due to the loosening of muscle fiber bundles after air thawing. In the UFT group, some muscle fibers were also broken under the influence of ultrasound. The degree of damage in the FT group was less than that in the UFT group. The fish in the MT group suffered from severe tissue water loss, increased muscle fiber exposure, and the most severe structural damage (Cao et al., 2019). In general, the LTT group maintained the quality of the samples better after thawing, while the MT group caused more damage to the sample quality.

Low temperature thawing well maintains the water retention, color and muscle composition of the samples, minimizes the oxidation of proteins and fats, and maintains the freshness of the samples. However, the longest thawing time was over 17 h. The microwave thawing rate was the fastest, but the myofibrils of the mackerel shrinked and broke, and the tail was matured, and the thawing loss rate and the cooking loss rate were the largest. Air thawing had little effect on the water retention, color and texture of the samples, but the prolonged contact with air resulted in high fat oxidation and high TVB-N content, which did not maintain the quality of mackerel well. The thawing speed of UFT is faster than FT, and after thawing, the difference between UFT and FT in terms of water holding power, color, texture, and TVB-N is smaller, which is because at 200 W power, the microscopic effect of ultrasonic waves on muscles is small and the damage to muscles is small. If this power is increased, the thawing speed of UFT will increase, but the quality deterioration of mackerel after thawing will be serious. If the power of ultrasound is reduced, the difference between the thawing result of UFT and FT will be smaller and ultrasound becomes meaningless. Regardless of time cost, low temperature thawing was the most ideal thawing method. The overall quality of mackerel after flow thawing was slightly lower than that of the low temperature thawing group, which was within the acceptable range, but it only took 1/43 of the latter's time.
